# Maternal Alexithymic Traits Are Related to Lower Maternal Sensitivity and Higher Hostility in Maternal Caregiving Behavior—The FinnBrain Birth Cohort Study

**DOI:** 10.3389/fpsyg.2021.704036

**Published:** 2021-07-26

**Authors:** Hanna Ahrnberg, Riikka Korja, Noora M. Scheinin, Saara Nolvi, Eeva-Leena Kataja, Jani Kajanoja, Hetti Hakanen, Linnea Karlsson, Hasse Karlsson, Max Karukivi

**Affiliations:** ^1^Department of Psychiatry, University of Turku and Turku University Hospital, Turku, Finland; ^2^FinnBrain Birth Cohort Study, Department of Clinical Medicine, Turku Brain and Mind Center, University of Turku, Turku, Finland; ^3^Department of Psychology and Speech Language Pathology, University of Turku, Turku, Finland; ^4^Turku Institute for Advanced Studies, University of Turku, Turku, Finland; ^5^Centre for Population Health Research, University of Turku and Turku University Hospital, Turku, Finland

**Keywords:** alexithymia, mother–infant interaction, maternal caregiving behavior, parenting behavior, sensitivity, non-hostility

## Abstract

**Background:** The quality of parental caregiving behavior with their child plays a key role in optimal mother–infant interaction and in supporting child adaptive development. Sensitive caregiving behavior, in turn, requires the ability to identify and understand emotions. Maternal alexithymia, with difficulties in identifying and describing feelings or emotions, as well as a concrete way of thinking, could potentially complicate the quality of caregiving. In this study, we aim to explore the possible association between maternal alexithymic traits and the quality of maternal caregiving behavior.

**Methods:** The study sample consisted of 158 mother–infant dyads within the FinnBrain Birth Cohort Study population with an available report of maternal alexithymic traits at 6 months postpartum and observational data on maternal caregiving behavior at 8 months postpartum. Alexithymia was measured using the 20-item Toronto Alexithymia Scale (TAS-20) including three alexithymia dimensions—Difficulty Identifying Feelings, Difficulty Describing Feelings (DDF), and Externally Oriented Thinking (EOT). Maternal caregiving behavior was assessed using the Emotional Availability Scale and in this study, all four parent dimensions (Sensitivity, Structuring, Non-intrusiveness and Non-hostility) were included. Maternal depressive and anxiety symptoms at 6 months postpartum were controlled for as potential confounders. In addition, background factors of mother's age and gestational weeks at the time of child birth, maternal educational level, monthly income and parity, as well as relationship status and the gender of the baby were assessed.

**Results:** Maternal TAS-20 total score correlated negatively with Sensitivity (*r* = −0.169, *p* = 0.034) and with non-intrusiveness (*r* = −0.182, *p* = 0.022). In addition, maternal DDF correlated negatively with Sensitivity (*r* = −0.168, *p* = 0.035) and EOT with Non-hostility (*r* = −0.159, *p* = 0.047). Furthermore, in regression analyses with controlling for the associated background factors, maternal total score of alexithymic traits (*p* = 0.034, η^2^*p* = 0.029) and higher DDF (*p* = 0.044, η^2^*p* = 0.026) remained significantly associated with lower Sensitivity and higher EOT remained significantly associated with lower Non-hostility (*p* = 0.030, η^2^*p* = 0.030).

**Conclusions:** In this explorative study we found preliminary evidence for the hypothesis that higher maternal alexithymic traits associate with lower maternal sensitivity and more hostile maternal caregiving behavior. Further studies are needed to explore these hypotheses and to investigate their possible implications for child development.

## Introduction

Well-functioning mother–infant interaction is reportedly beneficial for e.g., child socioemotional development, and optimal parent–infant interaction has been an interest of many scholars for decades (e.g., Emde, 1980; Bowlby, 1988; Biringen, 2008). The quality of caregiving behavior plays a key role in optimal mother–infant interaction. A child's ability to regulate emotions, attention and arousal develops in the context of their primary caregiving from the very beginning of infancy and thus, is dependent on bidirectional signaling and understanding of emotions (Bornstein et al., 2012). Sensitive caregiving behavior requires the ability to identify and understand emotions and thus, one potential factor that could complicate the quality of this primary caregiving is maternal alexithymia.

Alexithymia is a personality construct with a decreased capacity for identifying and expressing emotions, and restricted imaginary life (Taylor, 1984). It is most commonly assessed by the 20-item Toronto Alexithymia Scale, which measures alexithymia with three dimensions: Difficulty Identifying Feelings (DIF), Difficulty Describing Feelings (DDF) and Externally Oriented Thinking (EOT) (Bagby et al., 1994a,b). Parental alexithymia has been associated with impaired mother–infant relationship in dyads of mothers and their toddlers (Yürümez et al., 2014), and problems in parental mentalizing ability (Ahrnberg et al., 2020) as well as with weaker postnatal bonding (Ahrnberg et al. 2020, unpublished manuscript) at 6 months postpartum, in both mothers and fathers. However, to our knowledge, no studies examining the association between maternal alexithymic traits and observed maternal caregiving behavior exist.

In this study, we measured maternal caregiving quality by using the Emotional Availability construct that focuses on the caregiver's ability to regulate interactions through synchronous attunement to the child's affective and behavioral states (Emde, 2000). The Emotional Availability Scale (EAS, Biringen et al., 1998; Biringen, 2008) is an observational method developed to study both the parent's as well as the child's behavior in early mother–infant interaction (Biringen et al., 1998; Biringen, 2008). It includes four dimensions that measure parental caregiving behavior (Sensitivity, Structuring, Non-intrusiveness and Non-hostility). In general, studies using EAS have indicated the beneficial role of higher emotional availability in e.g., child socioemotional development, portrayed as the child's better expression of empathy toward adults (Moreno et al., 2008), better prosocial skills at 3–4 years of age (Howes and Hong, 2008), and better social skills and academic achievement through adolescence to adulthood (Raby et al., 2015).

In turn, common maternal postpartum psychiatric symptoms, such as depression and anxiety that are also associated with alexithymic traits (Honkalampi et al., 2000; Marchesi et al., 2005; Kajanoja et al., 2017), are known risk factors for poorer quality of maternal caregiving behavior. For instance, maternal depressive symptoms reportedly associate with more intrusiveness and withdrawal (Tronick and Reck, 2009) and less structuring parental behavior (Hakanen et al., 2019) as well as problems in parental emotion regulation (Riva Crugnola et al., 2016). Maternal depressive and anxiety symptoms are also linked to a less sensitive and responsive mother–infant interaction style (Ierardi et al., 2019). For instance, Hakanen et al. (2019) reported that increased maternal depressive symptoms were associated with child's lower involvement with the mother. Thus, given that alexithymia reportedly raises the risk for maternal psychiatric symptoms, these symptoms could be one mediating mechanism behind the poorer quality of maternal caregiving behavior.

To our knowledge, this is the very first study to examine the relationship between self-reported maternal alexithymic traits and observed maternal caregiving behavior. According to Bornstein et al. (2012), the question of which characteristics of the parent improve their ability to “read” the child's signals accurately and contribute to emotionally satisfying mother–infant interaction, is still unanswered, but a significant one. In this study, we aim to contribute to resolving this question by exploring whether there is an association between mother's alexithymic traits and maternal caregiving behavior after controlling for common postpartum psychiatric symptoms, anxiety and depression. More specifically, we aim to explore the unique associations between the different dimensions of alexithymia (DIF, DDF and EOT) and the four parent dimensions (Sensitivity, Structuring, Non-intrusiveness and Non-hostility) of the Emotional Availability Scale (Biringen et al., 1998; Biringen, 2008). We hypothesize that greater alexithymic traits at 6 months postpartum associate with poorer quality of maternal caregiving behavior at 8 months of the infant's age, as depicted by the EAS.

## Materials and Methods

### Procedure and Participants

This study was conducted within the larger FinnBrain Birth Cohort study (Karlsson et al., 2018). FinnBrain is a prospective birth cohort study that aims to investigate the influences of prenatal and early life stress on child health and development. Participants were recruited between December 2011 and April 2015 from maternal welfare clinics in the South-Western Hospital District and the Åland Islands in Finland. The study protocol was approved by the Ethics Committee of the Hospital District of Southwest Finland.

The study population of the current study consists of those 158 mother–infant dyads of whom the mother had filled in the 6-month questionnaire including the 20-item Toronto Alexithymia Scale and background factors including potential confounders, and who had participated in the mother–infant interaction observational assessment at the FinnBrain Child Development and Parental Functioning Study visit at 8 months postpartum. The 6-month questionnaire included also self-reported maternal depressive and anxiety symptoms that were included as potential confounders in this study. Other explored background factors were maternal age and gestational weeks at the time of childbirth, educational level, monthly income and parity, as well as mother's relationship status and gender of the baby.

Altogether 354 families were invited to participate in the mother–infant interaction observational study and 197 families participated. Seven families were excluded from this study due to inadequate data (*n* = 3) and four families for having fathers instead of mothers as participants, resulting in 190 families comprising the mother–infant interaction observation study population. From these 190 participants those mothers who did not return the 6-month questionnaire with the measure of alexithymic traits (*n* = 32) were further excluded from the study, thus leading to the final study population of the present study (*n* = 158).

Mothers who participated in the observational study were more likely to be highly educated and primiparous in comparison to those who were invited but chose not to participate (Hakanen et al., 2019). A more close description of the focus cohort and the recruitment process has been previously described by Hakanen et al. (2019). Those who did not return the 6-month questionnaire were more likely to have low education and lower age as well as more depressive and anxiety symptoms in previous measurements within the birth cohort study in comparison to those who returned the questionnaire at 6 months postpartum. A more detailed report of those who did and did not return the FinnBrain Birth Cohort's 6-month questionnaire has been previously given by Kajanoja et al. (2017). The cohort profile and the representativeness of the main cohort in comparison to general population has been described in more detail by Karlsson et al. (2018).

### Measures

#### Alexithymic Traits

Alexithymic traits were measured using a 20-item self-report questionnaire, the Toronto Alexithymia Scale (TAS-20). The 20 items comprise the following three dimensions: Difficulty in Identifying Feelings (DIF), Difficulty in Describing Feelings (DDF) and Externally Oriented Thinking (EOT). All items are rated on a 5-point Likert scale of one (strongly disagree) to five (strongly agree) with the total score ranging from 20 to 100 and with a greater score indicating a higher level of the alexithymic trait in question (Bagby et al., 1994a,b). TAS-20 is a widely used measure in different languages and cultures, and has been validated also in Finnish language (Joukamaa et al., 2001). It has been proven as a reliable and valid measure of alexithymic traits (Bagby et al., 2020).

For this study, the internal consistencies (Cronbach's alpha) for TAS-20 total score (α = 0.855) and DIF (α = 0.854) were good, acceptable for DDF (α = 0.779), and questionable for EOT (α = 0.636).

#### Observational Data on Maternal Caregiving Behavior

Maternal caregiving behavior was assessed using the Emotional Availability Scale (EAS) 4th Edition. EAS has been developed for observational studying of the parent–infant interaction by reflecting both the parent's as well as the child's perspectives (Biringen et al., 1998; Biringen, 2008), thus describing the overall quality of a parent–child relationship (Saunders et al., 2015). In this study, we included the four dimensions measuring the parent's perspective (Sensitivity, Structuring, Non-intrusiveness and Non-hostility). Sensitivity refers to the parent's ability to create and maintain positive and emotionally responsive behavior toward the child. Structuring reflects the parent's ability to support the child's individual learning processes by guiding and maintaining contact with the child. Non-intrusiveness refers to the parent's ability to let the child lead the play without the parent interfering, and to thus enhance the child's independence in actions, whereas Non-hostility refers to the parent's ability to regulate their own negative emotions during interaction (Biringen, 2008).

The procedure consisted of a 20-min free play mother–infant interaction that was video recorded in a laboratory setting. The mother and her infant were placed on a soft mat and provided with age-appropriate toys. The given instructions were: “This is a free-play time with your infant. You can use the toys, or you can play without the toys. Try to play with your infant as you normally would at home.” Assessments were conducted by a trained clinical psychologist or a psychology master student who did not take part in the interaction. The EAS coding was done by two blinded and reliable, trained coders. Each dimension ranges between scores from 1 to 7.

The internal consistencies were good for Sensitivity (α = 0.841) and Non-intrusiveness (α = 0.831), acceptable for Structuring (α = 0.765), and questionable for Non-hostility (α = 0.625) in this study. The inter-rater reliabilities (Cohen's kappa) were substantial for all the EAS dimensions: Sensitivity (0.80), Structuring (0.72), Non-intrusiveness (0.85) and Non-hostility (0.70).

#### Background Information and Potential Confounding Factors

Information on the background factors included maternal age (years), length of gestation at the time of childbirth and mother's parity (1 = primiparous and 2 = multiparous). In addition, the relationship status of the mother was explored (1 = married or in a registered relationship, 2 = not married, 3 = divorced or separated), as well as the gender of the baby (1 = girl, 2 = boy). As indicators of socioeconomic status, we included maternal educational level (1 = Low: high school, vocational degree or lower education; 2 = Middle: college degree or applied science degree; 3 = High: University education) and level of monthly income in euros divided into four different sub-classes (1 = Very low <1000 €; 2 = Low = 1001–2000 €; 3 = Middle = 2001–3000 €; 4 = High >3000 €).

Maternal postpartum depressive symptoms were measured at 6 months postpartum using the Finnish version of the Edinburgh Postnatal Depression Scale (EPDS; Cox et al., 1987; Tamminen, 1990). It is a widely used and a sensitive self-report measure for postnatal depressive symptoms. It consists of 10 items, each rated on a scale of 0–3 with a range of total score from 0 to 30 points. A higher score indicates more depressive symptoms (Cox et al., 1987). There are defined clinical cut-off points for possible depression (10–12) and possible severe depression (≥13) (Garcia-Esteve et al., 2003). In this study the EPDS score was used as a continuous variable in the final analyses because the study population consisted of general population where prevalence of probable depression was low, as expected. The internal consistency for EPDS in this study was good (α = 0.882).

Mother's anxiety symptoms were measured at 6 months postpartum with the anxiety subscale of the Finnish version of The Symptom Checklist 90 (SCL-90: Derogatis et al., 1973; Holi et al., 1998). It is a self-report questionnaire were the respondent is asked to report symptoms experienced within the last month. The anxiety subscale consists of 10 items with a 5-point scale that measures distress from 0 (not at all) to 4 (extremely), the total score thus ranging from 0 to 40 points, with a greater score indicating a higher level of anxiety symptoms. The clinical cut-off point for anxiety symptoms is usually set at > 9 points (Derogatis et al., 1973; Holi et al., 1998). As with depressive symptoms, the SCL-90 anxiety subscale was used as a continuous variable in the final models due to the limited number of mothers having clinical levels of anxiety symptoms. The questionnaire showed good internal consistency (α = 0.894).

### Statistical Methods

The normalities of the variable distributions were tested using Shapiro-Wilk test. Due to non-normal distribution of the variables, the association between continuous background factors and EAS dimension scores were studied using Spearman correlation coefficients, whereas Mann-Whitney U-test was used with dichotomous background factors and Kruskall-Wallis test with background factors of more than two categories. The significant bivariate associations between different TAS scores and EAS dimensions (Sensitivity, Non-intrusiveness and Non-hostility) were further examined using General Linear Model (GLM) in four different models with each EAS dimension as individual outcome variable. To ensure that GLM was appropriate to use even with non-normally distributed variables, the distribution of residuals of each model were explored and no violations against assumptions were detected. In each model, the potential effects of those background factors that were found to associate with the given EAS dimension were controlled for. Therefore, the models examining the association between Sensitivity and TAS-20 total score (Model 1) and Sensitivity and DDF (Model 2) were controlled for maternal educational level. In turn, the association between Non-hostility and EOT (Model 3) did not include any background factors, and the association between Non-intrusiveness and TAS-20 total score (Model 4) was controlled for maternal depressive symptoms, parity and educational level. The level of statistical significance was set at *p* < 0.05. The effect sizes were evaluated with partial eta squared with three levels: small (0.01), 0.09 (medium) and 0.25 (large). The statistical analyses were performed using SPSS (Version 24.0, IBM Corp, 2016).

## Results

### Descriptive Statistics

The descriptive statistics of the background variables, maternal alexithymic traits, and measures of maternal caregiving behavior are presented in Table 1. Information on educational level and monthly income was available for 156 participants. The majority had a middle (*n* = 64, 41.0%) or high level of education (*n* = 62, 39.7%) and only a minority had a low level of education (*n* = 30, 19.2%). Most of the incomes of the mothers fell within the low or mid-level of monthly income classes of this study population (low: 53.2%, *n* = 83 and middle: 28.8%, *n* = 45). Information on parity was available for 157 mothers, and 58.0% (*n* = 91) of the mothers in the sample were primiparous. The majority of mothers were married or in a registered relationship (*n* = 89, 56.3%), whereas 41.1% (*n* = 65) were not married and only 1.9% (*n* = 3) were divorced or separated. From one mother information on relationship status was not available. Most of the mothers (*n* = 130, 82.3%) did not have depressive symptoms, whereas 14 mothers (8.9%) exceeded the clinical cut-off points for possible depressive symptoms and equal amount (*n* = 14, 8.9%) for possible severe depressive symptoms. Similarly, most of the mothers (*n* = 140, 88.6%) did not suffer from anxiety symptoms whereas the minority, 11.4% (*n* = 18) exceeded the clinical cut-off point for possible anxiety symptoms. There were equal amount of boys and girls among the infants (*n* = 79, 50%).

**Table 1:** Descriptive statistics of maternal characteristics and alexithymic traits measured with the 20-item Toronto Alexithymia Scale (TAS-20) as well as mother-infant interaction measured with the Emotional Availability Scale.

**Background variables and alexithymic traits**	**Md (IQR)**	**n (%)**
*Educational level*		
Low		30 (19.2)
Middle		64 (41.0)
High		62 (39.7)
Monthly income		
Very low		24 (15.4)
Low		83 (53.2)
Middle		45 (28.8)
High		4 (2.6)
Parity		
Primiparous		91(58.0)
Multiparous		66 (42.0)
Relationship status		
Married or in a registered relationship		89 (56.3)
Not married		65 (41.1)
Divorced or separated		3 (1.9)
Gender of the baby		
Girl		79 (50)
Boy		79 (50)
Age	31.00 (5.00)	
Gwks	39.86 (1.71)	
SCL-90	2.00 (5.00)	
EPDS	4.00 (7.00)	
TAS-20 total score	39.00 (14.00)	
DIF	11.00 (7.00)	
DDF	9.00 (6.00)	
EOT	18.00 (6.00)	
*Emotional Availability Scale*		
Adult		
Sensitivity	5.50 (2.00)	
Structuring	5.00 (2.00)	
Non-intrusiveness	6.00 (2.50)	
Non-hostility	6.00 (2.00)	

*Gwks, gestational weeks; SCL-90, the Symptom Checklist 90 anxiety subscale; EPDS, the Edinburgh Postnatal Depression Scale; DIF, Difficulty Identifying Feelings; DDF, Difficulty Describing Feelings; EOT, Externally Oriented Thinking*.

### Associations Between Background Factors and Maternal Caregiving Behavior

First-time mothers (Mdn = 6.00, IQR = 3.00) were more intrusive (U = 3,911.00, *p* = 0.026) when compared to mothers with more than one child (Mdn = 6.00, IQR = 2.00). Higher educational level of the mother was associated with higher Sensitivity [H(2) = 8.649, *p* = 0.013] with median scores of 6.00 (IQR = 2.00) both for high and middle educational level and 5.00 (IQR = 2.50) for low educational level mothers. Higher educational level was also associated with less intrusive behavior in interaction [H(2) = 9.896, *p* = 0.007] with median scores of 6.00 within high (IQR = 2.00) and middle (IQR = 2.50) educational level categories and 5.00 (IQR = 2.5) within the low educational level mothers. Mothers relationship status and the gender of the baby were not significantly associated with any of the EAS dimensions.

Negative correlations were detected between maternal EPDS scores at 6 months and the EAS dimensions of Structuring (*r* = −0.157, *p* = 0.049) and Non-intrusiveness (r = −0.194, *p* = 0.015) at 8 months whereas SCL-90 anxiety symptoms at 6 months did not correlate with any of the EAS dimensions (see Table 2).

**Table 2:** Spearman correlations for parental dimensions of Emotional Availability Scale, alexithymic traits, depressive and anxiety symptoms as well as maternal age and gestational weeks at time of birth of the child.

	**1**	**2**	**3**	**4**	**5**	**6**	**7**	**8**	**9**	**10**	**11**
1 Sensitivity											
2 Structuring	0.704**										
3 Non-intrusiveness	0.481**	0.350**									
4 Non-hostility	0.595**	0.382**	0.478**								
5 TAS-20	−0.169*	0.126	−0.182*	−0.140							
6 DIF	−0.146	−0.117	−0.144	−0.111	0.789**						
7 DDF	−0.168*	−0.129	−0.132	−0.082	0.859**	0.629**					
8 EOT	−0.126	−0.103	−0.156	−0.159*	0.661**	0.205*	0.407**				
9 SCL-90	−0.117	−0.052	−0.154	−0.092	0.484**	0.616**	0.426**	0.696			
10 EPDS	−0.153	−0.157*	−0.194*	−0.150	0.536**	0.638**	0.461**	0.247	0.728**		
11 Age	0.037	0.128	0.062	0.033	−0.104	−0.085	−0.115	0.282	−0.185*	0.098	
12 Gwks	−0.74	−0.094	0.101	0.043	−0.005	0.003	−0.030	0.714	0.027	0.249	−0.093

* *p < 0.05*,

** *p < 0.01*.

*SE, Sensitivity; ST, Structuring; NI, Non-intrusiveness; NH, Non-hostility; TAS-20, The 20-item Toronto Alexithymia Scale; DIF, Difficulty Identifying Feelings; DDF, Difficulty Describing Feelings; EOT, Externally Oriented Thinking; SCL-90, The Symptom Checklist (Anxiety subscale); EPDS, Edinburgh Postpartum Depression Scale; Gwks, Gestational weeks*.

### Bivariate Associations Between Alexithymic Traits and Maternal Caregiving Behavior

The bivariate correlations between alexithymic traits at 6 months and maternal caregiving behavior at 8 months are displayed in Table 2. Maternal TAS-20 total score correlated negatively with Sensitivity (*r* = −0.169, *p* = 0.034) and with Non-intrusiveness (*r* = −0.182, *p* = 0.022). In addition, maternal DDF correlated negatively with Sensitivity (*r* = −0.168, *p* = 0.035) and EOT with Non-hostility (*r* = −0.159, *p* = 0.047).

### GLMs for the Alexithymic Traits at 6 Months Postpartum in Predicting Sensitivity, Non-intrusiveness and Non-hostility at 8 Months Postpartum

The general linear models for testing the association between alexithymic traits and the Emotional Availability Scale dimensions Sensitivity, Non-intrusiveness and Non-hostility, after controlling for background factors, are displayed in Table 3.

**Table 3:** Associations between the Emotional Availability Scales Sensitivity, Non-hostility and Non-intrusiveness and alexithymic traits with associated background factors and potential confounders explored by General Linear Model.

	***F***	***p***	**(***η^2^p)*****
**Model 1—Sensitivity**			
Corrected model predictor	5.340	0.002	0.095
TAS-20 total score	4.570	0.034	0.029
Educational level	4.060	0.019	0.051
*R*^2^ = 0.095, Adj. *R*^2^ = 0.077			
**Model 2—Sensitivity**			
Corrected model predictor	5.178	0.002	0.093
DDF	4.117	0.044	0.026
Educational level	4.026	0.020	0.050
*R*^2^ = 0.093, Adj. *R*^2^ = 0.075			
**Model 3—Non-hostility**			
Corrected model predictor	4.790	0.030	0.030
EOT	4.790	0.030	0.030
*R*^2^ = 0.030, Adj. *R*^2^ = 0.024			
**Model 4—Non-intrusiveness**			
Corrected model predictor	3.053	0.012	0.093
TAS-20 total score	0.460	0.499	0.003
EPDS	0.529	0.468	0.004
Parity	3.383	0.068	0.022
Educational level	3.668	0.028	0.047
*R*^2^ = 0.093, Adj. *R*^2^ = 0.062			

*TAS-20, the 20-item Toronto Alexithymia Scale; DDF, Difficulty Describing Feelings; EOT, Externally Oriented Thinking; EPDS, the Edinburgh Postnatal Depression Scale*.

The total TAS score of alexithymic traits was significantly negatively associated with Sensitivity (*p* = 0.034, η^2^*p* = 0.029) when controlling for maternal educational level (Model 1). Accordingly, the DDF dimension remained significantly negatively associated with Sensitivity (*p* = 0.044, η^2^*p* = 0.026) when controlling for maternal educational level (*p* = 0.020, η^2^*p* = 0.050) (Model 2).

EOT was significantly negatively associated with Non-hostility (*p* = 0.030, Model 3); however, no background or potential confounding factors were added in the model (see the Statistical methods for details).

## Discussion

To our knowledge, this study is the first to investigate the association between mothers' alexithymic traits and the quality of early maternal caregiving behavior. The main finding was that maternal alexithymic traits at 6 months postpartum were associated with maternal caregiving behavior in an observed free-play situation at 8 months postpartum. More in detail, higher scores of DDF (Difficulty Describing Feelings) were associated with lower maternal sensitivity, and higher scores of EOT (Externally Oriented Thinking) were significantly associated with more hostile behavior of the mother.

The construct of maternal sensitivity does not focus only on behavioral sensitivity (i.e., appropriate behavioral responses), but also on the appropriateness and awareness of the emotional climate of the mother–infant interaction (Easterbrooks et al., 2012). As alexithymic traits include difficulties in both identifying and describing feelings, it could be that mothers with alexithymic traits have difficulties in both creating and maintaining an optimal emotional climate within their interaction with their child. In addition, to be able to understand the affective cues of an infant, the mother must have the ability to see the infant as an individual person with his/her own feelings, needs and thoughts, meaning that the mother must have the ability for mentalizing, operationalized as reflective functioning (Fonagy and Target, 1997; Fonagy et al., 2012).

Our finding, indicating preliminary evidence for an association between alexithymic traits and problems in maternal caregiving behavior, is supported by our previous study indicating that stronger alexithymic traits of mothers were associated with weaker parental mentalizing ability (Ahrnberg et al., 2020). More specifically, the alexithymia dimensions EOT and DDF, that were associated with maternal caregiving behavior in this study, were also negatively correlated with parental reflective functioning factors “Interest in child mental states” as well as “Appropriate reasoning about child mental states” in the prior study. In addition, DDF was negatively correlated with the factor “Opacity of child mental states” in this previous study. However, this previous study also showed that when controlled for by educational level and depressive and anxiety symptoms, only EOT remained significantly associated with parental reflective functioning, while DDF did not (Ahrnberg et al., 2020). As a conclusion, it could be that maternal mentalizing ability, together with socio-economic factors and maternal psychiatric symptoms, may mediate the relationship between parental alexithymia and lower quality of maternal caregiving behavior. These hypotheses could be further tested in future studies by including mediation analyses.

In addition to the concepts described above, the parent's capacity to consider the motives underlying their infant's behavior and emotional experiences has been described as “insightfulness”, a conceptual cousin of mentalizing. This parental asset has also been identified as protective; Martinez-Torteya et al. (2018) presented in their study that insightful mothers showed high levels of positive parenting despite stressful life events. Thus, they suggest that enhancing insightfulness could have potential to buffer the mother–infant interaction from the effects of harmful stress. It has been presented that observational methods to evaluate maternal caregiving behavior could be complemented by methods, such as Insightfulness Assessment, in order to gain a more comprehensive assessment of maternal caregiving behavior (Oppenheim and Koren-Karie, 2013; Koren-Karie and Oppenheim, 2018).

The possible mechanisms underlying the association between maternal alexithymic traits and more hostile caregiving behavior seems more complex. It should also be acknowledged that hostility as a concept includes a wide range of hostile behaviors from concealed hostility to openly hostile responses (Biringen et al., 2014). Previous studies indicate that mothers with a history of depressive symptoms tend to present with hostile caregiving behavior more often than healthy controls (Sellers et al., 2014), as is also the case among mothers at high risk of developing postpartum depression (Frigerio et al., 2019). Alexithymic traits and higher risk of depressive symptoms are linked together with relatively strong evidence from the literature (e.g., Honkalampi et al., 2000; Kajanoja et al., 2017). However, EOT is not the alexithymia dimension often associated with depressive symptoms, whereas DIF and DDF are (Grabe et al., 2004; Conrad et al., 2009). There is hardly a straightforward explanation for the mechanism of association within this study either. However, it has been discussed whether EOT is associated with a specific unemotional cognitive style (Kajanoja et al., 2017) that could lead to a very concrete way of thinking and acting according to this. Possibly hostile caregiving behavior could act as a defensive way to react in overwhelming situations within the mother–child interaction for those mothers who score high especially in the dimension of EOT. The ability to see the child as a separate individual and with own emotions may have a role also within the association between alexithymic traits and hostility. An Australian study showed that mothers with better mentalizing ability were less hostile toward their 12-month old infants compared to mothers with weaker mentalizing abilities (Lok and McMahon, 2006). However, the underlying mechanism remains somewhat unexplained and these results should be interpreted with caution because the reliability of the measure for non-hostility is to some extent questionable.

The mother-infant interaction is potentially modifiable (Biringen et al., 2014). Thus, greater understanding of the mechanisms attached with possible risk factors of problematic caregiving behavior, such as alexithymic traits, could lead to planning intervention and prevention strategies. An important, yet complex open question is, whether parental alexithymic traits can be recognized in early stages of parenthood and furthermore enhance the emotional abilities through interventions. Interventions targeted in enhancing parental abilities, by e.g., increasing the sensitivity (Olds et al., 2002; Thomas and Zimmer-Gembeck, 2011; Alsancak-Akbulut et al., 2020) and diminishing the hostility (Naber et al., 2010) in parental caregiving behavior, have been successfully developed, but to our knowledge, not specifically tested or implemented within mothers having pronounced alexithymic traits.

To start with, better detection of mothers with alexithymic traits could provide a tool for preventive health care to find those mother–infant dyads at risk for suboptimal maternal caregiving behavior within the general population. The 20-item Toronto Alexithymia Scale is a relatively short questionnaire that is easy to administer and could be used, for example, in maternity clinics for the general population, for the identification of whom of the mothers are at risk for poorer caregiving behavior. Interventions in enhancing parenting behavior in general population have been developed as well. Alsancak-Akbulut et al. (2020) reported promising results on an intervention aiming to enhance maternal sensitivity among a general population sample. In their study, mothers with low educational level participating in a video-feedback intervention designed to enhance sensitive reactions of mothers toward their children showed an increase in sensitivity in comparison to controls (Alsancak-Akbulut et al., 2020). Considering mentalization as one possible mechanism explaining the link between alexithymic traits and maternal caregiving behavior, promising results have been reported about a mentalization-based program being beneficial preventive tool within primary health care services (Kalland et al., 2016; Sourander et al., 2021). However, research is lacking on whether the parenting behaviors of alexithymic mothers, in specific, could be enhanced with focused interventions. This matter calls for further studies.

As this is, to our knowledge, the first study to investigate the associations between mother's alexithymic traits and observed, professionally rated maternal caregiving behavior, further studies are needed. As one limitation, this study included only mother–infant pairs, whereas in the field of parenting research, more studies are nowadays focusing also on the fathers. It would be important to further develop this field of research to include triadic and family level perspectives. This is interesting also due to the greater prevalence of alexithymic traits among men than women. In general, the prevalence of alexithymic traits was lower within the Finnbrain birth cohort population in comparison to the reported prevalence in previous population studies (Franz et al., 2007; Mattila, 2009). This might be due to a selection bias with the participating couples expecting a baby and thus, being more engaged in to giving and receiving information on healthy child development and their own well-being.

Some limitations regarding the used measures should also be acknowledged. Alexithymia was measured only as a self-report and thus, the possibility of a reporting bias cannot be ruled out. However, mother–infant interaction was measured with an observational method thus increasing the overall objectivity of the results. In addition, more specific measures for the depressive and anxiety symptoms than the self-reports used in this study exists. However, the current study is a sub-study of a larger birth cohort which includes a wide variety of different measures selected based on usability within a larger scale. This also explains the given time points, e.g., different time points of measurement of alexithymic traits and observations of maternal caregiving behavior. Lastly, another limitation regarding the measures is that some of the dimensions of both TAS-20 and EAS showed only questionable internal consistencies. This should be noted especially regarding the model exploring the association between the EAS dimension Non-hostility and the alexithymia dimension EOT, as both of these dimensions had questionable internal consistencies, which may affect the reliability of these measures. With only modest effect sizes, the results of our study provide a preliminary basis for future studies. Concerning the statistical methods chosen, due to the exploratory nature of this study, we did not correct the *p*-values for multiple comparisons. In future studies the results of our study need to be replicated and especially if the number of statistical models should increase the need for multiple comparison is underlined.

Our study sample was a convenience sample meaning that those participating in the study were probably more likely to be interested and committed to gaining information about their parenting behavior. Thus, within this study population, both alexithymic traits as well as problematic maternal caregiving behavior were probably less prevalent than in the general population. However, in terms of exploring alexithymia that is not a personality disorder but a set of personality features, this kind of study population could be considered quite optimal with less confounding factors and comorbidity to control for. The strengths of this study include that the used measures were validated and internationally widely used. In the procedure of observation of maternal caregiving behavior, the coding was performed by two blinded and trained coders. Further, the sample size can be considered appropriate.

## Conclusions

Mother's alexithymic traits predicted less maternal sensitivity and more hostility toward the child in maternal caregiving behavior upon observation, when associated background factors were controlled for. To date, this is the first study to investigate the relationship between maternal alexithymic traits and observed maternal caregiving behavior with EAS, and therefore the underlying mechanisms, such as mentalizing capacities, as well as the significance of caregiving to mediate parental alexithymia influences on child outcomes should be further explored. The results of this study act as a preliminary basis for future studies. The clinical relevance is in aiming to detect those parental characteristics that could be used to screen and target interventions for those parents that especially need enhancement of parenting behavior.

## Data Availability Statement

The raw data supporting the conclusions of this article will be made available by the authors, without undue reservation.

## Ethics Statement

The studies involving human participants were reviewed and approved by the Ethics Committee of the Hospital District of Southwest Finland. Written informed consent to participate in this study was provided by the participants' legal guardian/next of kin.

## Author Contributions

HA: writing—original draft. RK and JK: writing—reviewing and editing. NS and MK: writing—reviewing and editing, supervision, and methodology. SN, E-LK, and HH: writing—reviewing and editing and investigation. LK and HK: resources, funding acquisition, conceptualization, and writing—reviewing and editing. All authors contributed to the article and approved the submitted version.

## Conflict of Interest

The authors declare that the research was conducted in the absence of any commercial or financial relationships that could be construed as a potential conflict of interest.

## Publisher's Note

All claims expressed in this article are solely those of the authors and do not necessarily represent those of their affiliated organizations, or those of the publisher, the editors and the reviewers. Any product that may be evaluated in this article, or claim that may be made by its manufacturer, is not guaranteed or endorsed by the publisher.
